# Total moderate-to-vigorous physical activity across the adult life course and the risk of invasive ovarian cancer

**DOI:** 10.1093/ije/dyag160

**Published:** 2026-08-28

**Authors:** Jennifer A Ritonja, Sreenath Madathil, Belinda Nicolau, Lisa Leung, Vikki Ho, Michal Abrahamowicz, Anita Koushik

**Affiliations:** Population Health Sciences, BC Cancer Research Institute, Vancouver, BC, Canada; Gerald Bronfman Department of Oncology, Faculty of Medicine and Health Sciences, McGill University, Montreal, QC, Canada; St. Mary’s Research Centre, Montreal, QC, Canada; Gerald Bronfman Department of Oncology, Faculty of Medicine and Health Sciences, McGill University, Montreal, QC, Canada; Faculty of Dental Medicine and Oral Health Sciences, McGill University, Montreal, QC, Canada; Gerald Bronfman Department of Oncology, Faculty of Medicine and Health Sciences, McGill University, Montreal, QC, Canada; Faculty of Dental Medicine and Oral Health Sciences, McGill University, Montreal, QC, Canada; Department of Epidemiology, Biostatistics and Occupational Health, Faculty of Medicine and Health Sciences, McGill University, Montreal, QC, Canada; Exposome and Heredity team, Inserm U1018, Center for Research in Epidemiology and Population Health (CESP), Gustave Roussy, Université Paris-Saclay, Villejuif, France; Université de Montréal Hospital Research Centre (CRCHUM), Montreal, QC, Canada; Department of Social and Preventive Medicine, Université de Montréal, Montreal, QC, Canada; Department of Epidemiology, Biostatistics and Occupational Health, Faculty of Medicine and Health Sciences, McGill University, Montreal, QC, Canada; Gerald Bronfman Department of Oncology, Faculty of Medicine and Health Sciences, McGill University, Montreal, QC, Canada; St. Mary’s Research Centre, Montreal, QC, Canada; Department of Epidemiology, Biostatistics and Occupational Health, Faculty of Medicine and Health Sciences, McGill University, Montreal, QC, Canada; Université de Montréal Hospital Research Centre (CRCHUM), Montreal, QC, Canada

**Keywords:** ovarian cancer, case–control study, physical activity, life-course epidemiology

## Abstract

**Background:**

Previous research on moderate-to-vigorous physical activity (MVPA) and ovarian cancer risk have shown inconsistent results. Most studies measured recreational MVPA once during late adulthood, which does not consider the way in which timing may differently influence risk or account for MVPA from other activities. Life-course epidemiology models (critical-period, sensitive-period, and accumulation) can evaluate whether the timing and cumulative burden of total MVPA across the lifespan influences risk. Here, we identify which model best describes the association between total MVPA, encompassing recreational, occupational, household, and transportation activities, and invasive ovarian cancer risk.

**Methods:**

A population-based case–control study of ovarian cancer in Montreal, Canada (2011–16) included 268 invasive cases and 668 controls aged ≥52 years. Total MVPA was determined by using recalled information on the frequency and duration of various physical activities across adulthood. A Bayesian relevant life course exposure model was used to estimate the relative importance of total MVPA, represented as average energy expenditure in units of metabolic equivalent (MET)-hours/week, during three pre-specified periods (early reproductive years, late reproductive years/perimenopause, and postmenopause) on invasive ovarian cancer risk.

**Results:**

The lifetime-effect odds ratio comparing ≥50 vs <50 MET-hours/week across adulthood was 0.59 (95% credible interval: 0.35–0.93). MVPA during late reproductive years/perimenopause showed the highest relative importance, suggesting that this may be a sensitive period. Associations were similar for serous and non-serous subtypes.

**Conclusion:**

Our results support that higher MVPA across the adult life course is associated with a reduced invasive ovarian cancer risk and mid-adulthood, represented by late reproductive years/perimenopause, may be a sensitive period.

Key Messages

- We used a life-course approach to examine the way in which the timing across adulthood of moderate-to-vigorous physical activity (MVPA) levels from all sources relates to invasive ovarian cancer risk.
- Our findings support that higher MVPA across the adult life course is associated with a reduced risk of invasive ovarian cancer and that late reproductive years/perimenopause may be a sensitive period.
- MVPA from multiple sources, i.e. not just recreation, and at certain periods in the adult life course may be particularly important for invasive ovarian cancer prevention.

## Introduction

In 2016, an estimated 28% of the global adult population did not meet the World Health Organization’s recommendation of ≥150 minutes per week of moderate-to-vigorous physical activity (MVPA) [1]. Higher MVPA reduces the risk of several cancers, yet evidence linking MVPA and ovarian cancer remains mixed [2]. Identifying modifiable factors for primary prevention is critical given that ovarian cancer is characterized by a poor prognosis and effective early detection methods are currently lacking [3].

Most epidemiological studies of MVPA and ovarian cancer assessed recreational activities once, generally during late adulthood [4–6], which does not capture other etiologically important life periods, nor account for MVPA from other domains. Physical activity levels can change throughout a woman’s life [7, 8], with notable shifts during the reproductive years, particularly from household activities [9, 10]. Thus, single-time-point assessments, particularly when close to diagnosis, and not considering all MVPA domains, i.e. recreation, occupation, household, and transportation, may miss etiologically important exposures. Many established ovarian cancer risk factors, particularly parity and oral contraceptive use, typically occur during early adulthood, supporting the importance of the timing of hormone-related exposures [11]. Given the hormone-sensitive nature of the ovary and that physical activity can influence endogenous sex hormones and ovulation [12, 13], it is biologically plausible that MVPA during specific life periods could be etiologically important, underscoring the need to consider exposure timing.

Life-course epidemiology provides a theoretical framework to enhance our understanding of ovarian cancer etiology by considering the timing of physical activity across different life periods [14]. In the present study, we aimed to identify the life-course hypothesis that best characterizes the association between total MVPA across adulthood and ovarian cancer risk, as supported by the data. We evaluated three alternative life-course hypotheses: (i) the accumulation hypothesis, which assumes that MVPA across all life periods contributes equally to risk, with the total lifetime effect being the accumulated effects across all periods; (ii) the critical-period hypothesis, which assumes that MVPA in only one period is important and has no effect outside this period; and (iii) the sensitive-period hypothesis, which assumes that MVPA during one or more periods has a disproportionately stronger effect on risk relative to the other periods [14].

## Methods

### Study population

The Prevention of OVArian Cancer in Québec (PROVAQ) study is a population-based case–control study that was conducted in Montreal, Canada (2011–16) [15] among women aged 18–79 years who were Canadian citizens residing in the Greater Montreal area. Newly diagnosed cases of primary borderline or invasive epithelial cancer of the ovary, fallopian tube, or peritoneum were identified from seven Montreal hospitals; 507 of 652 eligible cases (78%) participated, with 9 later excluded as non-epithelial or metastatic. Given the biologic, etiologic, and prognostic differences between invasive and borderline ovarian tumors, our analyses were restricted to the 364 invasive cancer cases. Controls were selected from the Quebec electoral list and frequency matched to cases by 5-year age group and Montreal region; 908 of 1634 eligible women (56%) agreed to participate. Through in-person interviews, data were collected on sociodemographic and lifestyle characteristics, medical history, lifetime occupational history, and various physical activities throughout adulthood.

### Assessment of domain-specific physical activities

The Supplementary materials provide details on the assessment and calculation of domain-specific MVPA. Briefly, participation in various recreational, occupational, household, and transportation activities was assessed from age 20 years to study participation, separately for winter and summer months. As previously described [16], recreational activities were determined from participants’ reports of regular participation in various moderate and vigorous leisure activities, including the activity type, time spent per week or month, and ages of participation, accounting for any breaks or changes in patterns over time. Occupational and transportation activities were determined from lifetime occupational histories, with participants reporting job titles, ages at which each job was held, status (part- or full-time), job tasks, and commuting time for various modes of transportation. Household activities were determined from reported time spent per week or month on gardening and general tasks (e.g. cleaning, child care), including any breaks or changes in patterns over time, and from occupational history information for periods as a “homemaker.”

### Calculation of total MVPA across the life periods

Similarly to previous work [16], we assigned activity-specific energy expenditures, in units of metabolic equivalents (METs), using the Compendium of Physical Activities [17]. For occupational activities, MET values were assigned to individual job tasks rather than to an overall job title by an expert occupational hygienist. Job-specific energy expenditure was calculated by weighting the task-specific METs by the estimated percentage of time spent on each task. The hygienist also assigned values for household activities for periods as a “homemaker” within the occupational history (see Supplementary materials). For all MVPA activities (i.e. those with ≥ 3 METs), MET-hours/week were calculated by multiplying the assigned MET value by the reported frequency and duration (in hours/week) for that activity, accounting for seasonal patterns. For each life period, domain-specific MVPA was then estimated by summing the MET‑hours/week for all activities within a given domain and dividing by the total years in that period to represent the average MVPA level over that period (Equation 1, Supplementary materials). The average total MVPA for each life period was then calculated as the sum of the four domain-specific averages. To reflect established stages in the adult female hormonal and reproductive life course and acknowledging evidence that early adulthood may be a particularly relevant period [18, 19], we a priori defined three life periods, consistent with our prior work [20]: (i) early reproductive years, (ii) later reproductive years/perimenopause, and (iii) postmenopause, represented by average total MVPA at ages 20–29, 30–49, and ≥50 years, respectively.

The most recent 2 years before diagnosis or interview (for controls) were excluded from the MVPA measure, to minimize potential reverse-causality bias and because recent exposures are unlikely to influence cancer development. To ensure that participants had MVPA information for each life period, we restricted our analysis to participants aged ≥52 years. Of the 364 invasive cases and 908 controls, 332 women aged ≤51 years and 6 women with missing MVPA were excluded, leaving 268 cases and 668 controls, representing 81% and 74% of each group, respectively.

Total MVPA was dichotomized by comparing ≥50 vs <50 MET-hours/week (equivalent to 3000 MET-minutes/week), consistent with the International Physical Activity Questionnaire definition of a “high” level [21, 22]. We selected this threshold for its public health relevance, as it represents a meaningful level of total MVPA accumulated across all domains [21, 22]. Studies using this threshold have reported greater risk reductions in chronic diseases relative to the typical minimum of 150 minutes/week [23, 24]. An individual can achieve 50 MET-hours/week through various daily physical activities; e.g. a sedentary worker could reach this goal through a combination of 30 minutes of food preparation and cooking, 20 minutes of cleaning with moderate effort, 15 minutes of running, and 30 minutes of walking for transportation. A continuous representation of total MVPA was also considered, but Bayesian Information Criterion comparisons of linear vs fractional polynomials did not support linearity for any life periods [25]. The dichotomous representation was therefore used for all analyses.

### Statistical analysis

We used the Bayesian Relevant Life Course Exposure Model (BRLM) to identify life periods in which total MVPA during adulthood may have the largest importance on invasive ovarian cancer risk [26]. Briefly, the BRLM estimates how the effect of an exposure accumulates over different life periods and allows for exposures during different life periods to contribute unequally to disease risk [26]. This is reflected in the exposure weights for each defined life period estimated in the BRLM, indicating, in our study, the relative importance of total MVPA during a given life period in relation to ovarian cancer risk. The weights are constrained between 0 and 1, and sum to 1 across all periods, and their variation reflects the life-course hypothesis best supported by the data. In particular: (i) for the life-accumulation model, all periods have equal weights; (ii) for the critical-period model, a weight of 1 is assigned to a single period, while all others are zero; and (iii) for the sensitive-period model, weights are nonzero for all life periods, but vary, with the highest weight corresponding to the sensitive period [26].

Using these estimated weights, the relevant life exposure is defined as the weighted sum of exposures across all life periods, where each life-period-specific (binary) exposure is multiplied by its corresponding weight [26]. The BRLM jointly estimates the life-period weights and the association between this weighted relevant life exposure and ovarian cancer risk in a single model via an unconditional logistic regression likelihood, providing an overall lifetime-effect odds ratio (OR). Because the weights are constrained to sum to 1, for binary exposures, a one-unit increase in the relevant life exposure represents the contrast between being exposed in all periods vs unexposed in all periods; thus, the lifetime-effect OR in our analysis is interpreted as the OR when comparing ≥50 vs <50 MET-hours/week across all periods.

We used a Hamiltonian Monte Carlo sampling approach to estimate posterior distributions for the exposure weights and the lifetime-effect OR. Four parallel Hamiltonian Monte Carlo chains were run for each analysis, where the first 25 000 iterations were considered for burn-in and the subsequent 50 000 iterations for inference. Convergence was assessed by using trace plots and Rhat values. Posterior estimates were summarized by using means and 95% credible intervals (CrIs). A non-informative Dirichlet (1, 1, 1) prior distribution was used for the weights (illustrated in Supplementary Figure 1 [26]), which assumes that all values for weights are equally plausible [26]. A weakly informative Student’s *t* (3, 0, 2.5) prior distribution was used for the lifetime-effect OR and covariate parameters [26]. The joint posterior distribution of weight estimates and CrIs were visualized by using ternary plots.

These analyses were conducted for invasive ovarian cancer overall and separately for serous and non-serous cancers. Additionally, we evaluated whether the association between lifetime total MVPA and ovarian cancer risk differed by the relative contributions of the activity domain. As occupational, household, and transportation activities are largely constrained by external circumstances [27, 28], recreational MVPA is the most modifiable domain for intervention. To capture this, we examined the association between total MVPA and ovarian cancer risk, stratifying by whether recreational activity comprised ≥25% or <25% of total MVPA (the median among controls), allowing us to distinguish participants with a greater vs lesser contribution of voluntary recreational activity to their total MVPA.

All models were adjusted for a minimal set of potential confounders identified by using a directed acyclic graph (DAG; Supplementary Figure 2), which included age, education, parity, oral contraceptive use, smoking status, lifetime alcohol intake, time spent outdoors, tubal ligation, and endometriosis. Missing data for confounders were minimal (<2%, see Table 1); thus, we used simple imputation of the median for continuous variables and mode for nominal variables, among controls.

**Table 1:** Characteristics of the PROVAQ study population aged ≥52 years.^a^

Characteristic	Controls (*N* = 668)	Cases (*N* = 268)
Age [mean (SD)]	63.9 (8.1)	64.1 (7.8)
Postmenopausal [*n* (%)]	573 (89.3)	240 (91.6)
Ancestry [*n* (%)]		
French Canadian	472 (70.8)	191 (71.3)
Other European ancestry	149 (22.3)	59 (22.0)
Other/mixed ancestry	46 (6.9)	18 (6.7)
Highest attained education level [*n* (%)]		
<High school	78 (11.7)	36 (13.4)
High school	167 (25.0)	79 (29.5)
College/technical	187 (28.0)	83 (31.0)
University or higher	236 (35.3)	70 (26.1)
Parity [*n* (%)]		
Nulliparous	134 (20.0)	68 (25.4)
1 or 2 births	378 (56.6)	154 (57.5)
≥3 births	156 (23.4)	46 (17.2)
Duration of oral contraceptive use [*n* (%)]		
Never	136 (20.4)	73 (27.2)
>0 to <10 years	380 (56.9)	155 (57.8)
≥10 years	152 (22.8)	40 (14.9)
Smoking status [*n* (%)]^a^		
Never	297 (44.5)	111 (41.4)
Former smoker	265 (39.7)	118 (44.0)
Current	106 (15.9)	39 (14.6)
Lifetime average alcohol intake [*n* (%)]^a^		
Never	175 (26.2)	91 (34.0)
>0 to <2 drinks/week	262 (39.2)	91 (34.0)
≥2 drinks/week	231 (34.6)	86 (32.1)
Lifetime average time spent outdoors in adulthood in h/week [mean (SD)]	6.6 (5.0)	6.8 (6.1)
Tubal ligation [*n* (%)]^a^		
Yes	217 (32.5)	77 (28.7)
No	451 (67.5)	191 (71.3)
Endometriosis [*n* (%)]^a^		
Yes	43 (6.4)	18 (6.7)
No	625 (93.6)	250 (93.3)
Family history of breast or ovarian cancer [*n* (%)]		
Yes	137 (21.0)	69 (26.0)
No	515 (79.0)	196 (74.0)
Body mass index in kg/m^2^ 2 years prior to diagnosis/study recruitment [mean (SD)]	26.4 (6.0)	26.9 (6.4)
Hormone-replacement use [*n* (%)]		
Ever	274 (41.4)	123 (46.2)
Never	388 (58.6)	143 (53.8)

a Missing information imputed for smoking status (1 case, 1 control), lifetime average alcohol intake (1 case, 4 controls), oral contraceptive use (2 cases), endometriosis (3 cases, 10 controls). Missing information not imputed for postmenopausal status (26 cases, 6 cases), ancestry (1 control), family history of cancer (16 controls, 3 cases who were adopted), body mass index (10 controls, 6 cases), and hormone-replacement use (6 controls, 2 cases).

Analyses were performed by using the “rstan” package to fit Bayesian models in R (R Version 4.5, R Foundation for Statistical Computing, Vienna, Austria).

## Results

The mean age of participants was 64.1 years (SD 7.8) for cases and 63.9 years (SD 8.1) for controls. Compared with controls, nulliparity and never drinking alcohol were more frequent among cases, while a university degree, oral contraceptive use, never smoking, and tubal ligation were less frequent (Table 1).

**Figure 1:**
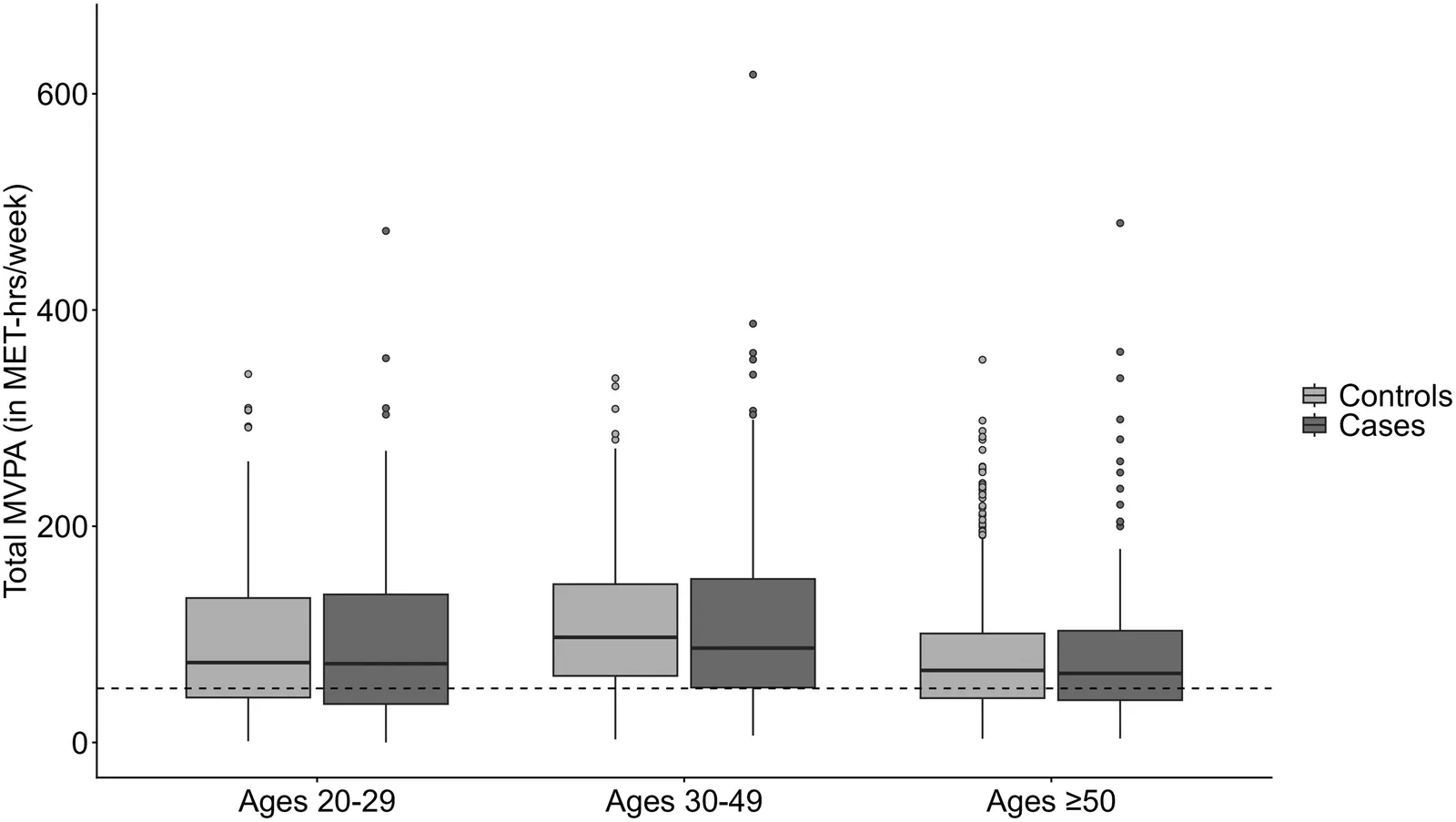
Box plot of total MVPA, by case–control status. Dotted line represents 50 MET-hours/week.

**Figure 2:**
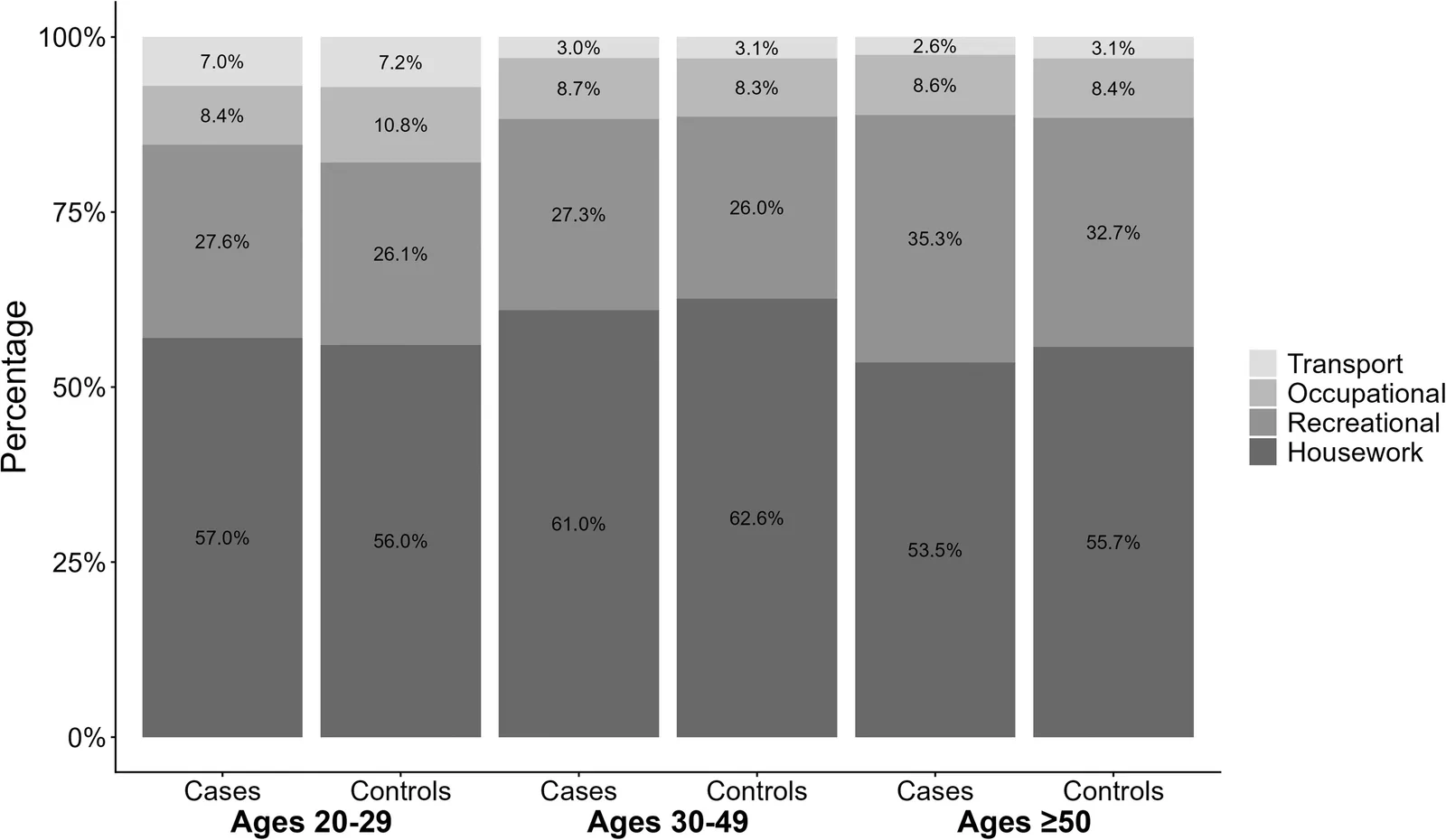
Mean proportion of total MVPA contributed by each domain across the life periods, by case–control status.

The median total MVPA across the adult life course was similar among cases and controls, with the highest levels during ages 30–49 years (Fig. 1). The MVPA values were moderately correlated between life periods (*r* = 0.36–0.67) (Supplementary Table 1). In all periods, household activities contributed the highest proportion to total MVPA (54%–63%), followed by recreational, occupational, and transportation (Fig. 2). The most common pattern of MVPA across the three periods was ≥50 MET-hours/week throughout adulthood (47% vs 50% of cases and controls,), followed by declining from ≥50 to <50 MET-hours/week after 50 years (11% vs 15% of cases and controls) (Supplementary Table 2). More cases than controls stayed at <50 MET-hours/week across the life course (18% vs 10%, respectively).

**Figure 3:**
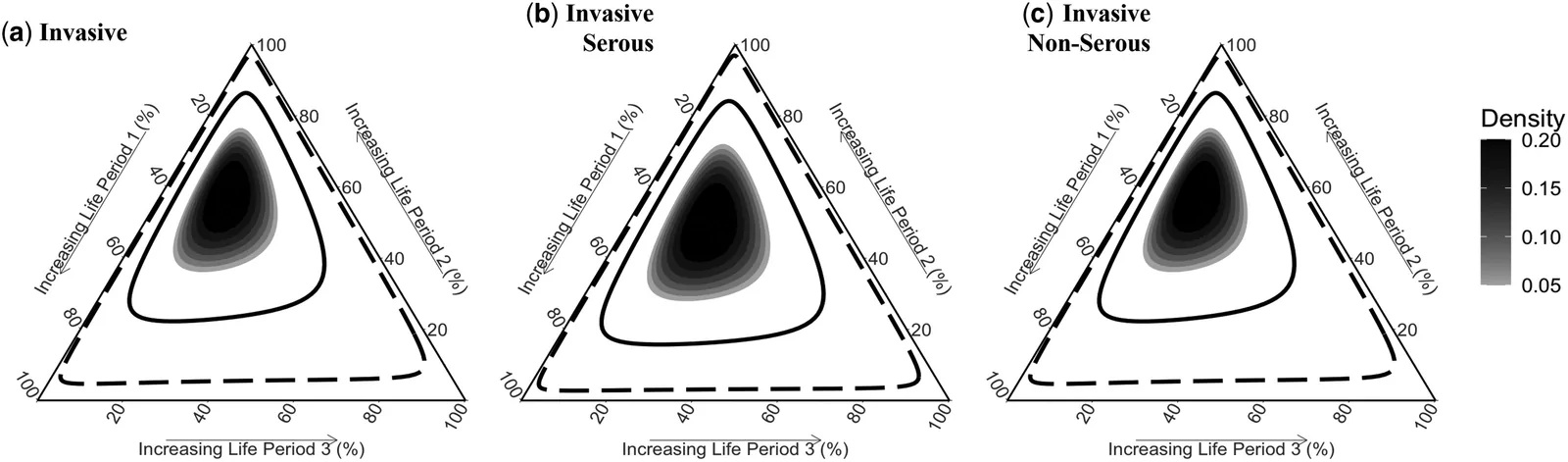
Densities and credible limits of posterior joint distributions of weights for the relationship between total MVPA (<50 vs ≥50 MET-hours/week) during the three life periods and (a) invasive, (b) invasive serous, and (c) invasive non-serous ovarian cancer risk. Solid and dashed lines represent 50% and 95% credible intervals, respectively. Darker areas represent higher posterior densities, where density concentrated near the center supports the accumulation hypothesis, density concentrated near one or more vertices supports the critical-period hypothesis, and density concentrated elsewhere supports the sensitive-period hypothesis.

The overall lifetime-effect OR for invasive ovarian cancer when comparing ≥50 vs <50 MET-hours/week was 0.59 (95% CrI: 0.35–0.93) (Table 2). The posterior probability for an OR of <1.0 was 99%, strongly supporting a reduced risk with higher lifetime MVPA (Table 2). The mean weights (i.e. relative importance) for the three life periods differed and was highest for ages 30–49 years (mean weight = 0.52, vs 0.29 and 0.19 for ages 20–29 and ≥50 years, respectively), supporting late reproductive years/perimenopause as a sensitive period (Table 2 and Fig. 3; posterior probability for a sensitive period 68%). Results were similar for serous and non-serous types (Table 2 and Fig. 3).

**Table 2:** Associations^a^ between life-course total MVPA (comparing ≥50 vs <50 MET-hours/week) and invasive ovarian cancer risk, overall and for serous and non-serous types.

	Mean weight^b^ (95% CrI)	OR (95% CrI)	Posterior probability for a sensitive period^c^ (%)	Posterior probability for OR < 1 (%)
**Invasive ovarian cancer (268 cases, 668 controls)**				
Ages 20–29 years	0.29 (0.01–0.74)		23.0	
Ages 30–49 years	0.52 (0.06–0.90)		67.7	
Ages ≥50 years	0.19 (0.01–0.59)		9.3	
Lifetime effect		0.59 (0.35–0.93)		98.7
**Invasive serous ovarian cancer (206 cases, 668 controls)**				
Ages 20–29 years	0.32 (0.02–0.79)		29.5	
Ages 30–49 years	0.46 (0.03–0.89)		56.4	
Ages ≥50 years	0.22 (0.01–0.68)		14.1	
Lifetime effect		0.65 (0.36–1.07)		95.8
**Invasive non-serous ovarian cancer (62 cases, 668 controls)**				
Ages 20–29 years	0.29 (0.01–0.76)		26.0	
Ages 30–49 years	0.44 (0.03–0.88)		52.9	
Ages ≥50 years	0.27 (0.01–0.73)		21.2	
Lifetime effect		0.51 (0.20–1.04)		96.9

a Adjusted for age (continuous), education (<high school, high school, college/technical, undergraduate and postgraduate), parity (nulliparous, 1 or 2, ≥3 births), oral contraceptive use (never, >0 to <10, ≥10 years), smoking status (current, former, never smoker), lifetime average alcohol intake (never, >0 to <2, ≥2 drinks/week), average time spent outdoors across adulthood (continuous), tubal ligation (yes, no), and endometriosis (yes, no).

b Mean weights refer to the relative importance of total MVPA during a given life period in relation to ovarian cancer risk.

c Refers to the posterior probability that a given sensitive-period hypothesis (e.g. for ages 20–29 years, the probability that the weight parameter for ages 20–29 years > the weight parameter for ages 30–49 years and the weight parameter for ages ≥50 years) is supported by the data.

Among women whose recreational activities accounted for ≥25% of their total MVPA, the lifetime-effect OR (0.52, 95% CrI: 0.26–0.94) and weight for ages 30–49 years (mean weight = 0.55) were both slightly stronger than those for the full population (Table 3). Conversely, for those with <25%, the lifetime-effect OR was supportive of a weaker risk reduction (0.79, 95% CrI: 0.40–1.38; posterior probability for OR < 1.0 = 82%) and the mean weights for the three life periods were more similar to each other, ranging from 0.29 to 0.37 (Table 3).

**Table 3:** Associations^a^ between life-course total MVPA (comparing ≥50 vs <50 MET-hours/week) and invasive ovarian cancer risk, stratified by the proportion of total MVPA that is from recreational activities.

	Mean weight^b^ (95% CrI)	OR (95% CrI)	Posterior probability for a sensitive period^c^ (%)	Posterior probability for OR < 1 (%)
**Recreational activities accounted for <25% of total MVPA (133 cases, 341 controls)**				
Ages 20–29 years	0.37 (0.02–0.85)		39.8	
Ages 30–49 years	0.34 (0.01–0.83)		34.8	
Ages ≥50 years	0.29 (0.01–0.79)		25.4	
Lifetime effect		0.79 (0.40–1.38)		82.1
**Recreational activities accounted for ≥25% of total MVPA (135 cases, 327 controls)**				
Ages 20–29 years	0.21 (0.01–0.63)		11.9	
Ages 30–49 years	0.55 (0.07–0.91)		72.8	
Ages ≥50 years	0.24 (0.01–0.68)		15.3	
Lifetime effect		0.52 (0.26–0.94)		98.4

a Adjusted for age (continuous), education (<high school, high school, college/technical, undergraduate and postgraduate), parity (nulliparous, 1 or 2, ≥3 births), oral contraceptive use (never, >0 to <10, ≥10 years), smoking status (current, former, never smoker), lifetime average alcohol intake (never, >0 to <2, ≥2 drinks/week), average time spent outdoors across adulthood (continuous), tubal ligation (yes, no), and endometriosis (yes, no).

b Mean weights refer to the relative importance of total MVPA during a given life period in relation to ovarian cancer risk.

c Refers to the posterior probability that a given sensitive-period hypothesis (e.g. for ages 20–29 years, the probability that the weight parameter for ages 20–29 years > the weight parameter for ages 30–49 years and the weight parameter for ages ≥50 years) is supported by the data.

## Discussion

This study identified that the sensitive-period life-course hypothesis best characterizes the association between total MVPA across the adult life course and invasive ovarian cancer risk. In particular, our findings support an inverse association, with the late reproductive years/perimenopause being a sensitive period. Stronger risk reductions were observed when recreational activity accounted for a greater proportion of total MVPA, highlighting its potential relevance for intervention.

Over 35 studies have examined MVPA in relation to ovarian cancer risk, with inconsistent findings [4–6]. While some assessed MVPA at multiple time points [29–33], most relied on a single assessment [4–6]. Our study improved upon this by using a life-course framework and showed that accounting for the dynamic nature of MVPA while estimating the relative importance of specific life periods is important. For example, similarly to studies examining MVPA closer to diagnosis [4–6], if we used only a single measure of average MVPA at ages of ≥50 years, a conventional logistic regression model would estimate an OR of 0.90 [95% confidence interval (CI: 0.65–1.25)], failing to indicate the inverse association revealed in our BRLM analyses.

Inconsistent findings in previous research may also reflect variation in the MVPA domains assessed, though most focused solely on recreational MVPA [4–6]. Our prior analysis of lifetime MVPA from recreational sources only showed a null association with invasive ovarian cancer among postmenopausal participants [16], in contrast to the inverse association observed here when using total MVPA. Only 11 previous studies assessed other domains [29–39], of which only one study included all four, assessed once close to diagnosis [34], reporting an inverse association (OR: 0.49; 95% CI: 0.35–0.68 for >23 vs <12 MET-hours/week) [34]. Two others examined recreational plus transportation domains, with mixed results [30, 39], while eight assessed recreational plus household and/or occupational activities (including homemaker tasks), reporting either a reduced risk [32, 33, 35, 36], increased risk [29, 37], or null association [31, 38]. These inconsistencies may partly reflect differences in how domestic activities were defined, as many lacked details on child-care tasks. Our study showed a higher proportion of household activities vs other domains compared with other studies [27, 29, 37], likely because we included child-care tasks when assessing household MVPA. Interestingly, among five studies assessing MVPA at two or more time points (regardless of domain) [29–33], all but one [29] reported an inverse association. Overall, these findings underscore the importance of assessing MVPA across (i) all domains, particularly household activities; and (ii) different life periods.

Nonetheless, recreational MVPA remains most amenable to intervention and thus the most promising target for prevention, given that occupational, household, and transportation activities are often constrained by external factors (e.g. job requirements, domestic responsibilities) and feasibility [28]. In analyses stratified by the proportion of total MVPA that was recreational, the inverse lifetime effect of total MVPA, as well as the relative importance of the sensitive period in late reproductive/perimenopausal years, was stronger among individuals with higher (≥25%) recreational MVPA. This supports the promotion of recreational MVPA for prevention, particularly in mid-adulthood.

Part of the association between MVPA and ovarian cancer risk is likely mediated through reductions in adiposity [40], though MVPA has adiposity-independent anticancer actions, including modulating inflammation, ovulation, endogenous sex hormones, and immune function [41, 42]. Hormonal fluctuations and continued ovulation during late reproductive and perimenopausal years may promote a pro-inflammatory environment and stimulate ovarian epithelial cell proliferation [42]. MVPA in this period may both reduce initiating tumorigenesis and suppress the progression of oncogenic processes initiated earlier [43, 44], as observed in experimental models of ovarian and other tumors [45, 46]. These broad biological effects may explain why associations were similar for serous and non-serous cancers.

Given that the recall of physical activities covered the entire adult lifetime, some error is inevitable. We used a life-events calendar to assist recall [47] and our questionnaire was similar to those of other studies examining recalled lifetime MVPA and cancer risk [48, 49]. Measurement error in lifetime MVPA is inherently complex, given that recall error is likely to be greater for more distant exposures, thus it is possible that the relative importance of early reproductive years was attenuated. Nevertheless, we believe that the degree of attenuation is unlikely to be large enough to have altered our overall posterior conclusion supporting a sensitive period in later reproductive years and perimenopause. Further, there is some evidence that recall errors in case–control studies do not explain the inverse associations between lifetime physical activity and cancer risk [50]. Selection bias is also possible, although adjustment for factors such as education, smoking, alcohol, and parity, which are likely associated with both participation and MVPA [51], may have partly mitigated its impact [52]. In particular, information collected from women who refused to participate showed that participants tended to be younger, had higher education, and smoked less [15]. Finally, while our study is strengthened by the use of a DAG to identify possible confounders, unmeasured confounding is possible given the limited understanding of invasive ovarian cancer etiology.

In conclusion, our findings support the sensitive-period hypothesis, with higher MVPA across adulthood associated with reduced invasive ovarian cancer risk and the late reproductive and perimenopausal years emerging as a potential sensitive period. In addition, the benefits of total MVPA may increase if a larger proportion comes from recreational sources. While more research is needed to further understand the way in which MVPA during different life periods may affect ovarian cancer risk, our findings offer novel insights that could inform tailored prevention strategies.

## Supplementary Material

dyag160_Supplementary_Data

## Data Availability

The data that support the findings of our study are available from the corresponding author upon reasonable request and institutional approval. Computing code that supports the findings of this study is publicly available at osf.io/xmnsb.
