# Supplementary material for: Total moderate-to-vigorous physical activity across the adult life course and the risk of invasive ovarian cancer
Source: Int J Epidemiol. 2026 Aug 28;55(5):dyag160. doi: 10.1093/ije/dyag160 (PMC13524439; doi:10.1093/ije/dyag160)
Supplement: Supplementary file 1 [file dyag160_supplementary_data.pdf]

SUPPLEMENTARY MATERIALS

Total moderate-to-vigorous physical activity across the adult life course and the risk of invasive ovarian cancer

Jennifer A. Ritonja[1,2,3], Sreenath Madathil[2,4], Belinda Nicolau[2,4,5], Lisa Leung[6], Vikki Ho[7,8], Michal Abrahamowicz[5], Anita Koushik[2,3,5,7]

Affiliations:

1. Population Health Sciences, BC Cancer Research Institute, Vancouver, BC, Canada
2. Gerald Bronfman Department of Oncology, Faculty of Medicine and Health Sciences, McGill University, Montreal, QC, Canada
3. St. Mary's Research Centre, Montreal, QC, Canada
4. Faculty of Dental Medicine and Oral Health Sciences, McGill University, Montreal, QC, Canada
5. Department of Epidemiology, Biostatistics and Occupational Health, Faculty of Medicine and Health Sciences, McGill University, Montreal, QC, Canada
6. Exposome and Heredity team, Inserm U1018, Center for Research in Epidemiology and Population Health (CESP), Gustave Roussy, Université Paris-Saclay, Villejuif, France
7. Université de Montréal Hospital Research Centre (CRCHUM), Montreal, QC, Canada
8. Department of Social and Preventive Medicine, Université de Montréal, Montreal, QC, Canada

Table of Contents

Calculation of MVPA 2

Supplementary Tables and Figures 7

Supplementary Figure 1. Densities and credible prior limits of the Dirichlet prior distribution (1,1,1) for the weights. 7

Supplementary Figure 2. Directed acyclic graph for the association between adult lifetime total moderate-to-vigorous physical activity (MVPA) and ovarian cancer risk. 8

Supplementary Table 1. Correlation matrix of total moderate-to-vigorous physical activity (MVPA, in MET-hrs/week) during different life periods 9

Supplementary Table 2. Patterns of total moderate-to-vigorous physical activity (MVPA) over the adult life course, by case-control status. 10

Calculation of MVPA

General formula for calculation of total moderate-to-vigorous physical activity (MVPA)

We estimated MVPA similar to previous work.[1] The general formula used to calculate MVPA in each of the four domains is described here, with exact details provided below for each domain. We first assigned activity-specific energy expenditures using the Compendium of Physical Activities,[2] which provides standardized metabolic equivalents (MET) values for a wide range of physical activities. For all MVPA activities (i.e., those with MET ≥3), MET-hrs/week were calculated by multiplying the assigned MET value by the reported frequency and duration (in hours/week) for that activity, accounting for seasonal patterns. For each life period, domain-specific MVPA was then estimated by summing the MET-hrs/week for all activities within a given domain and dividing by the total years in that period to represent the average MVPA level over that period (see Equation 1). Average total MVPA for each life period was then calculated as the sum of the four domain-specific averages.

$$\frac{\sum\frac{[(Year_{End}-Year_{Start}+1)](Winter\ months/year)(4.33\ weeks/month)(Days/week)(Hrs/day)(MET_{Activity-Winte}\ )}{52\ weeks/year}}{\#\ Years\ in\ life\ period} + \frac{\sum\frac{[(Year_{End}-Year_{Start}+1)](Summer\ months/year)(4.33\ weeks/month)(Days/week)(Hrs/day)(MET_{Activity-Summer})}{52\ weeks/year}}{\#\ Years\ in\ life\ period} \quad \text{Equation (1)}$$

Where **$Year_{End}$** and **$Year_{Start}$** indicated when each activity began and ended; **Winter or Summer months/year** indicated the number of months the activity occurred for by season; **4.33 weeks/month** was used as the average number of weeks per month; **Days/week** reflected how many days per week the activity was performed; **Hrs/day** indicated the number of hours per day; and **$MET_{Activity}$** represented the MET value assigned to each activity.

Occupational MVPA

To determine occupational MVPA, participants' job histories were evaluated by an occupational hygienist. For each job, the hygienist evaluated the job title (coded using the International Standard Classification of Occupations [ISCO] 1968),[3] and the reported tasks for each job (up to three tasks reported for each job). Each task was assigned a MET value using the Adult Compendium of Physical Activities. Additionally, the hygienist estimated the percentage of time spent on each task based on their expertise. Examples of occupations with only low MET values (≤1.5) included secretaries, accountants, and occupations related to

management and administration, whereas jobs that spent the majority of time in moderate-to-vigorous MET values (≥3.0) included emergency services (e.g., police officers), cleaners, fitness instructors, and dancers.

Occupational MVPA was then calculated by summing the MET values of all job tasks, weighted by the proportion of time spent on each task, using Equation 1. We assumed full-time jobs were 5 days/week at 8 hours/day, part-time jobs were 2.5 days/week at 8 hours/day, and volunteer 'jobs' were 1 day/week for 2.5 hours, aligning with Canadian averages.[4,5] Occupations listed as 'homemaker, 'student', or 'retired' were not considered as part of occupational MVPA and not included in the calculations. No participants had missing data on occupational history.

Transportation MVPA

When recalling occupational histories, for each reported job, participants provided the modes of transportation and total number of minutes per day, round-trip, spent commuting to work during winter months (September to May) and summer months (June to August). Modes of transportation assessed included walking, jogging, type of public transport (i.e., bus, metro or train), driving, bicycling, or other transport (e.g., motorcycle, scooter). Only walking, jogging and bicycling were considered MVPA (i.e., MET≥3.0):

List of moderate and vigorous physical activities for transportation:

Activity	Compendium Activity Code[2]	Metabolic Equivalents (MET) Value
Walking	16060	3.5
Bicycling	01011	6.8
Jogging	12020	7.0

For each participant, we calculated average transportation MVPA in units of MET-hrs/week using Equation 1. Similar to occupational MVPA, we assumed full-time jobs were 5 days/week, part-time jobs were 2.5 days/week, and volunteer 'jobs' 1 day/week. If a participant reported using public transport (bus, metro or train) but did not additionally report walking, bicycling or driving to a transit station, we additionally added 20 minutes of walking to their transport MVPA, which reflects the average distance a Greater Montreal resident lives from a transit stop (~500m).[6] Similar to occupational MVPA, we did not include transportation times for occupations listed as 'homemaker, 'student', or 'retired'.

One participant (covering two jobs) reported a mode of transport but no corresponding travel time, and thus we imputed the missing time using the median reported by other participants using that mode. Nineteen participants (across 31 jobs) were missing mode of transport. Because the median MET value for commuting in our study population was 1.3 (reflecting bus, car, or train

travel) and therefore sedentary, we assumed these missing transport activities were also sedentary. Since our analysis focused on MVPA, and these activities did not meet the ≥3 MET threshold, they were not included in our MVPA calculations.

Recreational MVPA

Calculation of recreational MVPA has been previously described in detail.[1] Briefly, participants reported details of regular participation in moderate and vigorous recreational activities conducted during their leisure time during adulthood. A full list of activities is provided in the original publication,[1] and included activities such as walking/hiking, jogging or running, bicycling, aerobic exercise, golf, swimming or water sports, skiing or skating, team or individual sports and other strenuous exercise. For each reported activity, participants provided the start and end ages, the number of hours per week or month spent in the activity during different seasons, and any breaks or changes in patterns over time. We calculated average recreational MVPA for all activities ≥3.0 MET in units of MET-hrs/week using Equation 1.

As reported previously,[1] when participants reported participating in an activity but the frequency was missing, we imputed the frequency using the season-specific 25th percentile among participants who reported that activity. Among >19,000 activity periods reported in the full study population, 1.3% had missing frequencies.

Household MVPA

We estimated household-related MVPA separately for periods when household work was vs. was not the primary occupational activity:

For periods when primary occupational activity was outside the household, (i.e., employed, graduate school, retired), we estimated household-related MVPA using reported details of regular participation in housework activities during adulthood using two questions: 1) Reported participation in general household activities, which encompassed various domestic activities including cooking, cleaning, laundry, childcare, and more; and 2) reported participation in gardening (similar to other validated MVPA questionnaires[7]). For both, participants provided the start and end ages, the number of hours per week or month spent in the activity during different seasons, and any breaks or changes in patterns over time. Energy expenditure for gardening was assigned a MET of 3.8 (Compendium Code: 08245).[2] Given activities for various domestic duties were captured within a single question, we assumed that 50% of their reported time was spent in moderate or vigorous activities based on population averages for such tasks, and assigned a MET of 3.4. This corresponds with the simple average between the two activities of childcare and cleaning that likely represented the majority of participant's time in MVPA based on these population averages, as shown in the table below. We then calculated average household MVPA in units of MET-hrs/week using Equation 1.

When participants reported participating in general household activities or gardening but the frequency was missing, we imputed the frequency using the season-specific 25th percentile among participants who reported that activity. Among >3,500

household activity periods reported in the full study population, 0.1% had missing frequencies, and among >1,200 gardening activity periods, 1.2% had missing frequencies.

Summary of literature-derived population averages for time spent in various household physical activities:

Activity Type	Population Average (in mins/day)	Compendium Activity Code[2]	Compendium Activity Description	Metabolic Equivalents (MET) Value
**Moderate or vigorous physical activities (≥3 MET)**				
Taking care of child/baby	30-60[10-13]	05186	Child care, standing (e.g., dressing, bathing, grooming, feeding, occasional lifting of child), moderate effort	3.5
Cleaning house, general	30-60[12,14]	05030	Cleaning, house or cabin, general, moderate effort	3.3
Yard work	5[14]	08261	Yard work, general, moderate effort	4.0
		08262	Yard work, general, vigorous effort	6.0
Total	65-125			
**Light physical activities (1.5<MET<3)**				
Food preparation	50-55[12,14]	05050	Cooking or food preparation – standing or sitting or in general (not broken into stand/walk components), manual appliances, light effort	2.0
Laundry	15[14]	05090	Laundry, fold or hang clothes, put clothes in washer or dryer, packing suitcase, washing clothes by hand, implied standing, light effort	2.0
Light household chores (e.g., maintenance, repairs)	10-25[14]	05025	Multiple household tasks all at once, light effort	2.8
Total	75-95			

For periods when primary occupational activity was within the household, (i.e., ‘homemaker’, ‘stay-at-home parent’), household-related MVPA was estimated using reported information (tasks) provided in their occupational histories. The occupational hygienist evaluated task descriptions for each reported task/role (including childcare responsibilities when specified, which accounted for number of children) and assigned MET values to the reported activities using the Adult Compendium of Physical Activities. Household-related MVPA was then calculated in MET-hrs/week using Equation 1. We assumed household activities occurred for 8-hours/day for 7 days/week, similar to other physical activity questionnaires.[8,9] For these periods, we excluded overlapping household physical activity derived from the questionnaire, with the exception of gardening, which we also calculated across adulthood as described above. No participants had missing data on occupational history.

References

1. Grundy A, Ho V, Abrahamowicz M, et al. Lifetime recreational moderate-to-vigorous physical activity and ovarian cancer risk: A case–control study. Int J Cancer. 2020;146(7):1800-1809. https://doi.org/10.1002/ijc.32513
2. Ainsworth BE, Haskell WL, Herrmann SD, et al. 2011 Compendium of Physical Activities: a second update of codes and MET values. Med Sci Sports Exerc. Aug 2011;43(8):1575-81. https://doi.org/10.1249/MSS.0b013e31821ece12
3. International Labour Organization. (1968). International standard classification of occupations, Revised, 1968.
4. Statistics Canada. Table 14-10-0320-02: Average usual hours and wages by selected characteristics, monthly, unadjusted for seasonality. https://doi.org/10.25318/1410032001-eng
5. Statistics Canada. Table 45-10-0039-01: Volunteer rate and average annual volunteer hours, by definition of volunteering and age group. https://doi.org/10.25318/4510003901
6. El-Geneidy A, Grimsrud M, Wasfi R, Tétreault P, & Surprenant-Legault J. (2014). New evidence on walking distances to transit stops: Identifying redundancies and gaps using variable service areas. Transportation, 41(1), 193-210. https://doi.org/10.1007/s11116-013-9508-z
7. Craig CL, Marshall AL, Sjöström M, et al. International physical activity questionnaire: 12-country reliability and validity. Med Sci Sports Exerc. 2003;35(8):1381-1395. https://doi.org/10.1249/01.MSS.0000078924.61453.FB
8. Friedenreich CM, Courneya KS, Bryant HE. Reliability and validity of the Past Year Total Physical Activity Questionnaire. Am J Epidemiol. 2006;163(10):959-970. https://doi.org/10.1093/aje/kwj112
9. Saint-Maurice PF, Berrigan D, Whitfield GP, et al. Amount, Type, and Timing of Domain-Specific Moderate to Vigorous Physical Activity Among US Adults. J Phys Act Health. 2021;18(S1):S114-S122. https://doi.org/10.1123/jpah.2021-0174
10. Dong L, Block G, Mandel S. Activities Contributing to Total Energy Expenditure in the United States: Results from the NHAPS Study. Int J Behav Nutr Phys Act. 2004;1(1):4. https://doi.org/10.1186/1479-5868-1-4.
11. US Bureau of Labor Statistics. 2025. American Time Use Survey News Release. Accessed from: https://www.bls.gov/news.release/atus.htm.
12. Statistics Canada. 2015. Average time spent per day on various activities, for the population and participants aged 15 and over, by sex, Canada, 2010. Accessed from: https://www150.statcan.gc.ca/n1/pub/89-647-x/2011001/tbl/tbl11-eng.htm.
13. Statistics Canada. 2024. Table 45-10-0104-01: Daily average time spent on various activities, by age group and gender, 2022. https://doi.org/10.25318/4510010401-eng.
14. US Bureau of Labor Statistics. 2025. Average hours per day spent in selected household activities. https://www.bls.gov/charts/american-time-use/activity-by-hldh.htm

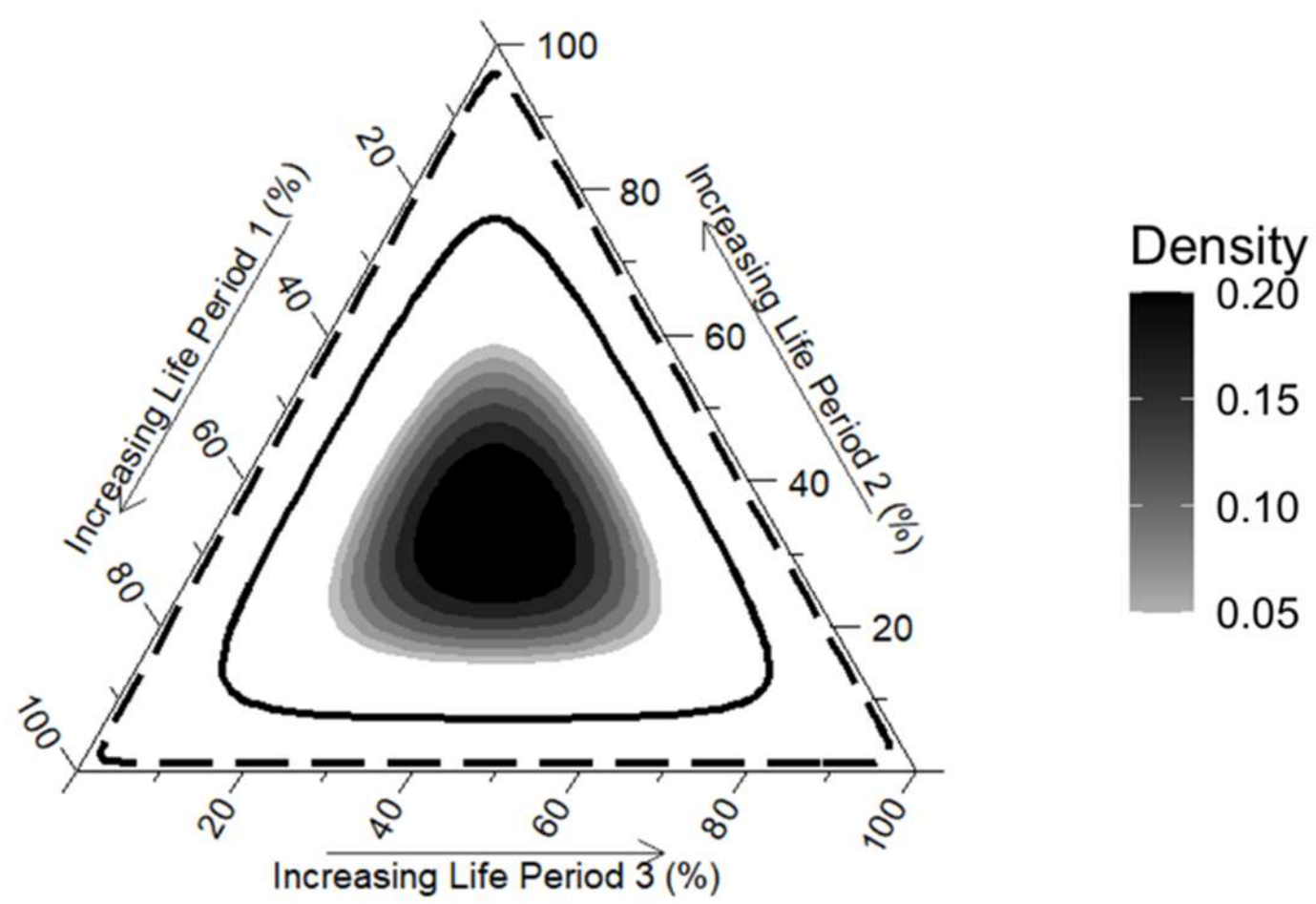

Supplementary Figure 1. Densities and credible prior limits of the Dirichlet prior distribution (1,1,1) for the weights. Solid and dashed lines represent 50% and 95% credible intervals, respectively. Darker areas represent higher posterior densities, where density concentrated near the center supports the accumulation hypothesis, density concentrated near one or more vertices supports the critical period hypothesis, and density concentrated elsewhere supports the sensitive period hypothesis.

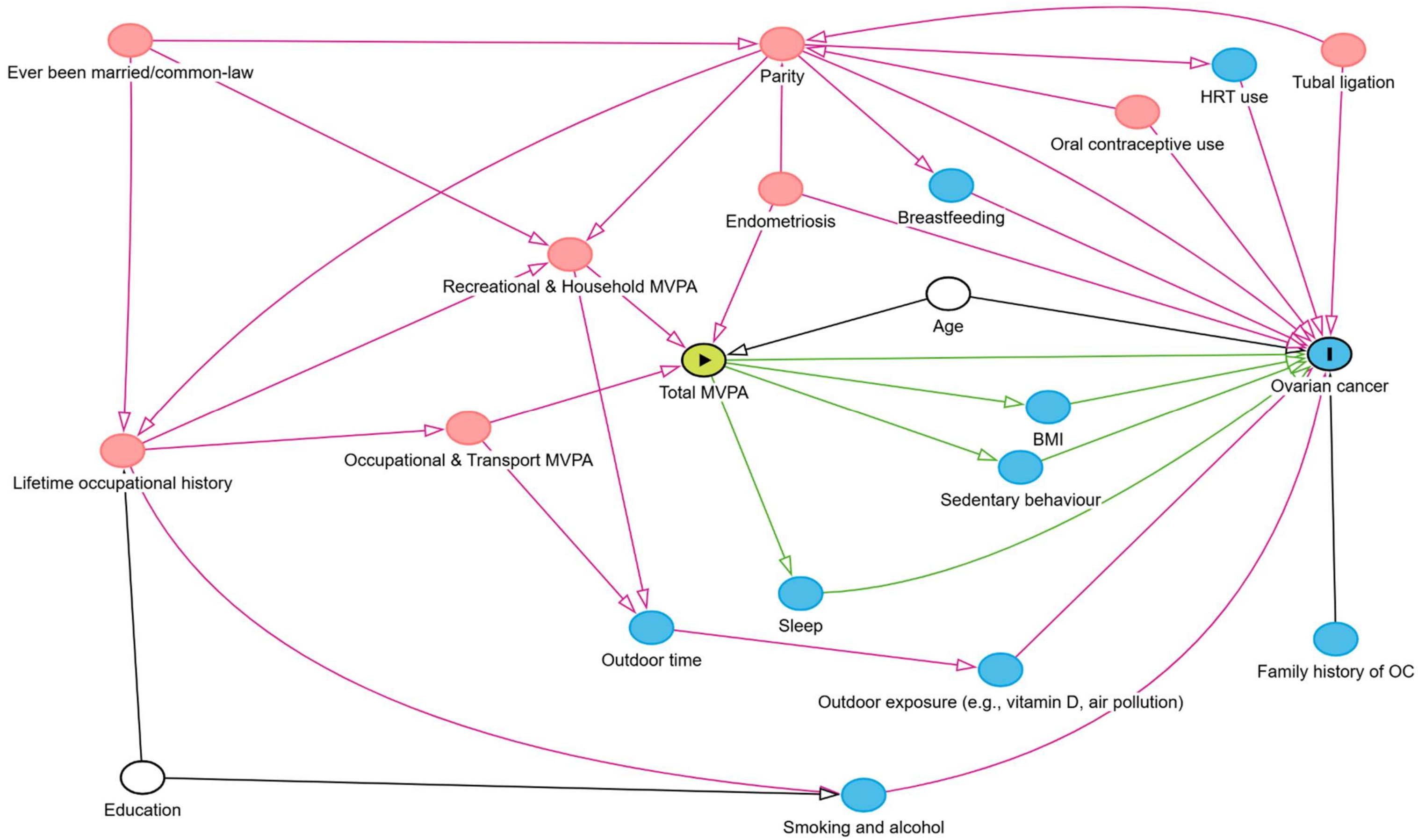

Supplementary Figure 2. Directed acyclic graph for the association between adult lifetime total moderate-to-vigorous physical activity (MVPA) and ovarian cancer risk. We selected the most parsimonious minimally sufficient set of adjustment variables which included age (frequency-matching variable), highest level of education attained, parity, oral contraceptive use, smoking status, lifetime average alcohol intake, average time spent outdoors across adulthood, tubal ligation, and endometriosis.

Supplementary Table 1. Correlation matrix of total moderate-to-vigorous physical activity (MVPA, in MET-hrs/week) during different life periods.

**Ages 20-29**		
0.63	**Ages 30-49**	
0.36	0.67	**Ages ≥50**

Supplementary Table 2. Patterns of total moderate-to-vigorous physical activity (MVPA) over the adult life course, by case-control status.

Life course total MVPA			N Controls (%)	N Cases (%)
Ages 20-29	Ages 30-49	≥Ages 50		
0	0	0	67 (10.0)	47 (17.5)
0	0	1	23 (3.4)	7 (2.6)
0	1	0	43 (6.4)	12 (4.5)
0	1	1	82 (12.3)	35 (13.1)
1	0	0	16 (2.4)	9 (3.4)
1	0	1	7 (1.0)	4 (1.5)
1	1	0	99 (14.8)	29 (10.8)
1	1	1	331 (49.6)	125 (46.6)

Note: Life course total MVPA were <50 MET-hrs/week (0) or ≥50 MET-hrs/week (1).
